# Advances in Airway Management and Ventilation Strategies in Emergency Medicine

**DOI:** 10.1155/2015/425715

**Published:** 2015-06-24

**Authors:** Tomasz Gaszynski, Kamil Toker, Massimiliano Carassiti, Athanasios Chalkias, Jestin N. Carlson

**Affiliations:** ^1^Department of Emergency Medicine and Disaster Medicine, Medical University of Lodz, Lodz, Poland; ^2^Department of Anaesthesia and Reanimation, Kocaeli University Hospital, Umuttepe, Kocaeli, Turkey; ^3^School of Medicine, Campus Bio-Medico University, Rome, Italy; ^4^MSc “Cardiopulmonary Resuscitation”, Medical School of University of Athens, Athens, Greece; ^5^Hellenic Society of Cardiopulmonary Resuscitation, Athens, Greece; ^6^Allegheny Health Network, Department of Emergency Medicine, Saint Vincent Hospital, USA

Effective management of the airway is a priority in resuscitation efforts and a central issue in emergency medicine for providing ventilation and oxygenation in critically ill patients. Compared to the controlled conditions of the operating theatre, airway management in the emergency department is more difficult than in other circumstances; the patient may be in respiratory distress, desaturating, or may have a compromised airway. In addition, sedation may have a profound effect on hemodynamically unstable patients, who may become severely hypotensive and rapidly deteriorated, while preparation of the patient, environment, and equipment is often challenging [1, 2].

This special issue is published with the intent of serving as a procedure manual for disseminating advances in airway management and ventilation strategies to all providers who are involved in emergency care. This issue highlights the explosive growth of video laryngoscopy which is rapidly becoming first-line for both in- and out-of-hospital airway management.

In this issue, S. Fujiwara et al. compare the utility of the Pentax-AWS Airwayscope (AWS) with the GlideScope (GS) during chest compressions on an infant manikin. They report that the AWS performs better than the GS for endotracheal intubation during ongoing cardiopulmonary resuscitation (CPR).

S. Lee et al. compare intubation performances among Pentax-AWS (AWS), GlideScope (GVL), and Macintosh laryngoscope (MCL) during mechanical chest compression in 15° and 30° left lateral tilt, simulating maternal cardiopulmonary resuscitation. Their study indicates AWS as an appropriate laryngoscope for airway management of pregnant women in lateral tilt.

P. Schoettker and J. Corniche assess the quality and speed of intubation between the Airtraq with its new iPhone AirView app and the King Vision in a manikin study. They report that the Airtraq-AirView allows faster identification of the landmarks and intubation in a difficult airway manikin, highlighting the need for further research. Also, H. Y. Choi et al. acknowledge the importance of early airway management in severely ill patients by investigating the efficacy of face-to-face intubation in four different types of laryngoscopes. In this study, Pentax Airway Scope and Macintosh were the most favorable laryngoscopes in face-to-face intubation.

In an extensive review, M. Barak et al. discuss the complexity and difficulties of securing the airway of patients with maxillofacial trauma and present their approach for airway management of such patients. Despite the recent advances in emergency airway management techniques, healthcare personnel may still face the “cannot ventilate, cannot intubate” scenario in which the specialized equipment may be invaluable. In this context, T. Doi et al. investigated the influence of upper airway resistance (UAR) during transtracheal jet ventilation by comparing a manual jet ventilator (MJV) and the oxygen flush device of the anesthetic machine (AM). In their model, the influence of choked flow from the Venturi effect was minimal under all UAR settings with the MJV, but the AM could not deliver sufficient flow.

J. N. Carlson et al. perform a proof of concept study to determine if portable motion technology could identify the motion components of endotracheal intubation between novice and experienced providers using inertial measurement units (IMUs) to record the movements during endotracheal intubation. They conclude that portable IMUs can be used to detect differences in movement patterns between novice and experienced providers, suggesting their value in educational efforts.

Field intubation is a complex process and time to intubation, number of attempts, and hypoxia have all been shown to correlate with increases in morbidity and mortality. B. Boehringer et al. investigate the “Impact of Video Laryngoscopy on Advanced Airway Management by Critical Care Transport Paramedics and Nurses Using the CMAC Pocket Monitor” in field intubation. They report that the CMAC video laryngoscope improves success rates in airway management, indicating that video laryngoscopes may be crucial in out-of-hospital airway management.

As many challenges are augmented in the acute setting, emergency providers must be skilled with airway management [3]. Advances in airway management technology have helped to improve many aspects of emergency airway management; however, expertise alone cannot make up for the lack of the right equipment or adequate understanding of new technologies. Conversely, these new technologies do not obviate the need for a solid foundation in airway management techniques. Up to 4% of patients who undergo emergent intubation suffer a cardiac arrest [4], indicating that a more complete understanding of the interaction between clinical experience and technological advances is needed to increase the effectiveness and improve the safety of patients undergoing emergency airway management. This special issue helps to expand our knowledge of this intersection within emergency airway management.

*Tomasz Gaszynski* *Kamil Toker* *Massimiliano Carassiti* *Athanasios Chalkias* *Jestin N. Carlson*
